# Delayed diagnosis of an atypical rupture of an unscarred uterus due to assisted fundal pressure: a case report

**DOI:** 10.1186/1757-1626-2-7966

**Published:** 2009-06-03

**Authors:** Mertihan Kurdoglu, Ali Kolusari, Recep Yildizhan, Ertan Adali, Hanim Guler Sahin

**Affiliations:** Department of Obstetrics and Gynecology, Yuzuncu Yil University School of MedicineVanTurkey

## Abstract

**Introduction:**

Although rare, rupture of an unscarred uterus is one of the most dangerous obstetric complications, resulting in maternal and fetal jeopardy.

**Case presentation:**

A 30-year-old grand multiparous Turkish woman without any history of uterine surgery gave birth vaginally at 37 weeks of gestation with fundal pressure applied in the second stage of labor. Transabdominal sonography performed 32 hours after delivery due to postural hypotension and a drop in hemoglobin values in the postpartum period revealed massive intra-abdominal free fluid. On emergency laparotomy, serosal rupture of the uterus on the left posterior side was observed. She underwent a subtotal hysterectomy and did well postoperatively.

**Conclusion:**

Postural hypotension in postpartum patients without any evident vaginal bleeding may be an early sign of possible uterine rupture, even if the vital signs are stable. Early diagnosis is important if maternal morbidity and mortality are to be decreased.

## Introduction

Uterine rupture is a rare but serious complication in obstetrical practice. Cesarean section is the most important predisposing factor for this catastrophic event and it is usually reported during labor in patients with such a scarred uterus. Uterine rupture in an unscarred uterus is seen much more rarely, with an estimated occurrence of one in 8000-15000 deliveries [1]. Beside cesarean section, inappropriate prostaglandin and oxytocin usage, previous instrumental abortion, vacuum extraction delivery, and vigorous fundal pressure are the other risk factors for uterine rupture [1,2]. These cases are usually diagnosed intrapartum or shortly after delivery and managed with immediate repair of the usually encountered full-thickness rupture site or subtotal hysterectomy. Here we present a patient with a ruptured unscarred uterus who was diagnosed 32 hours after delivery. The rupture was different from those usually seen and was thought to be due to assisted fundal pressure during the second phase of labor.

## Case presentation

A 30-year-old gravida 5, para 4, abortion 0 Turkish woman was admitted for spontaneous labor at 37 weeks' gestation. She had experienced normal spontaneous vaginal deliveries for her first four pregnancies and had undergone no uterine surgery. On admission, her blood pressure was 120/80 mmHg and pulse was 110 beats/min. Cervical dilatation was 5 cm and cervical effacement 40%. The membranes were intact and the presentation was vertex at floating station. Initial cardiotocographic monitoring showed a normal fetal heart rate with good variability and accelerations. No decelerations were present. Labor contractions were at 2-3 minute intervals with high pressure tension.

During this stage of labor, neither oxytocin nor prostaglandin augmentation was administered. Following an uneventful labor, 5 hours after admission a vaginal examination revealed a completely dilated cervix and 0 to +1 station. The patient was encouraged to push the baby. When the fetus reached a +1 to +2 station, decelerations with every contraction were seen on the fetal monitor. The mother was immediately taken to the labor room for a rapid trial vaginal delivery. Since she was too exhausted to push the fetus properly, assisted fundal pressure was applied. After a series of applications of assisted fundal pressure, a female infant weighing 3200 g was born with the cord wrapped around her neck three times. Apgar score was 4 at 1 minute and 6 at 5 minutes. There was no meconium in the amniotic fluid.

**Figure 1.**
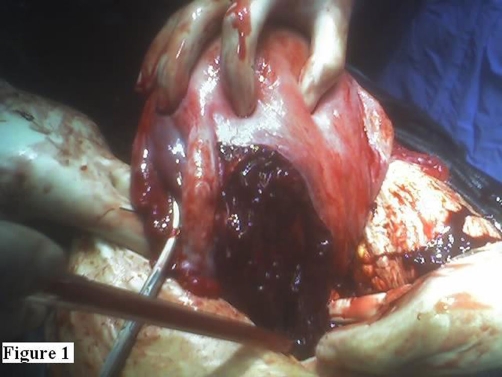
A large left broad ligament hematoma with multiple small bleeding points from the branches of the uterine artery is seen.

**Figure 2.**
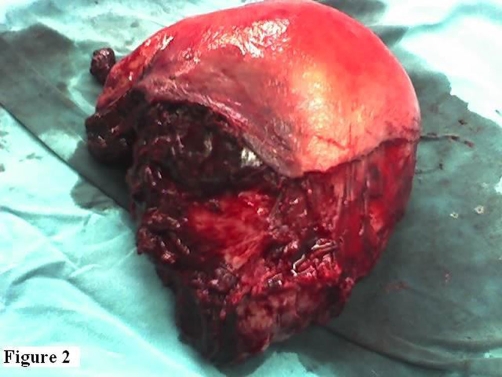
Subtotal hysterectomy material is shown.

Other than a sudden drop in maternal blood pressure to 80/50 mm-Hg and then a rise to normal values, the immediate postpartum period was uneventful. Although vital signs of the patient were stable during her follow-up, about 8 hours after delivery postural hypotension was noted. At that time, the hemoglobin (Hg) value was found to have dropped to 6.5 g/dl from an initial value of 10.7 g/dl on admission. In the postpartum period, no abnormal vaginal bleeding was observed. This was attributed to bleeding during delivery and 2 units of complete blood were transfused. Since 6 hours and 24 hours later Hg values were found to be 9.3 g/dl and 4.9 g/dl, respectively, transabdominal sonography was performed and massive intra-abdominal free fluid was observed. An emergency laparotomy was performed and 1000 cc hemoperitoneum with a large vertical tear on the left posterior side beginning from just above the insertion of left uterosacral ligament was identified. The tear extended into one-third of the uterine wall. Thus, the uterine and peritoneal cavities were not communicating. A large left broad ligament hematoma with multiple small bleeding points from the branches of uterine artery was also present (Figure 1). A repair could not be performed due to tissue edema and friability of the myometrium. A subtotal hysterectomy was performed (Figure 2) and three additional units of complete blood were transfused perioperatively. Her postoperative condition was stable. The patient was discharged home together with her healthy baby 4 days later.

## Discussion

Uterine rupture is one of the most important obstetric emergencies, threatening the lives of both mother and fetus. There are two types of rupture: 1) complete, where the whole thickness of the uterine wall is involved, usually occurring in an unscarred uterus; and 2) incomplete, where the visceral peritoneum remains intact, as seen in scar dehiscence [3]. We could not categorize our case using either definition since it was somewhat different. The rupture seen in our case was not complete because only one-third of the uterine wall was involved, without communication between the uterine and the peritoneal cavities. We think that this may be the reason why excessive bleeding through the vaginal route was not seen. The rupture was not incomplete either, since the visceral peritoneum was not intact at that site. Although most cases in the literature were placed into one of these two categories, only a few were similar to ours in appearance. Langton et al reported a case of spontaneous uterine rupture that occurred in a nonlaboring uterus of a primigravid with no previous risk factors at 32 weeks and a tear extending into two-thirds of the uterine wall with small actively bleeding vessels was identified during laparotomy [4]. The patient in our case was a grandmultiparous woman at term and laboring.

The most common presentation is intrapartum, but rupture can be diagnosed ante- or postpartum. Intrapartum events are usually detected after a sudden increase in maternal pulse rate and a decrease in blood pressure together with vaginal bleeding and abdominal pain followed by fetal bradycardia [5]. However, in the postpartum period, a clinical diagnosis is difficult and a high index of suspicion is essential. Hruska et al described a patient who presented to the outpatient obstetrical clinic with worsening left lower quadrant abdominal pain 4 days postpartum of an uncomplicated, vaginal delivery involving low dose oxytocin stimulation and underwent a total abdominal hysterectomy due to a defect in the left lower uterine wall [6]. That patient stated that the pain had been present since after her delivery but had worsened in the past day and described the pain as a “burning” sensation radiating to her left back. In our case, the only positive signs in the patient were a transient drop in blood pressure during labor and orthostatic hypotension afterwards. The patient did not report any specific type of pain other than some pain thought to be related to parturition. However, the patient did not want to undergo surgery for some time when it was recommended after our preoperative diagnosis of uterine rupture.

Risk factors for uterine rupture include obstructed labor, grand multiparity, placenta percreta, induction of labor in a woman with a previously scared uterus, uterine anomalies, inappropriate prostaglandin and oxytocin usage, previous instrumental abortion, vacuum extraction and forceps delivery, vigorous fundal pressure, and intrauterine manipulations [1,2]. In our patient, the identified risk factors were grand multiparity and fundal pressure.

Fundal pressure is the application of steady pressure on the fundus of the uterus and is considered among the most controversial maneuvers that are used in the second stage of labor since no confirmed benefit of the procedure has been documented and a few adverse events have been reported in association with its use.

Pan et al reported a case of uterine rupture due to traumatic fundal pressure in a primigravid woman with an unscarred uterus [1]. Wei et al also reported an unusual case associated with traumatic assisted fundal pressure related uterine rupture at 34 weeks of gestation in a pregnancy complicated by hydrops fetalis and shoulder dystocia [7]. In a review by Güney et al, 1 out of 8 ruptured unscarred uterus cases was found to be related to the application of fundal pressure [8]. A prospective study consisting of 63 patients with uterine rupture, among whom half had a uterine scar, revealed that fundal pressure, along with forceps and oxytocin use, was an iatrogenic factor associated with uterine rupture [9]. However, in that study, there was no control group and so it is unknown whether fundal pressure was an independent factor in causing uterine rupture [10]. In our case, the mother had an unscarred uterus and was not given any prostaglandin or oxytocin during labor. We applied assisted uterine fundal pressure during the delivery since fetal bradycardia was noted and the mother was too exhausted to push the baby properly. Thus, we tried to accelerate the second stage of labor in order to save the baby. However, Wei et al stated that they applied fundal pressure because of coexistent shoulder dystocia [7], but fundal pressure should be avoided if shoulder dystocia is identified. If applied in this circumstance, the shoulder will be further impacted and increase the chances of injury to the baby [11]. In this situation, fundal pressure possibly also increases the risk of uterine rupture.

## Conclusion

Assisted fundal pressure during delivery can result in trauma even to the unscarred uterus and cause traumatic uterine ruptures. During the postpartum period, the patients with risk factors having stable vital signs and postural hypotension without any evident vaginal bleeding should be promptly examined for possible uterine rupture. Early diagnosis is vital if maternal morbidity is to be decreased.

## Abbreviations

None.
